# Natural reservoirs for homologs of hepatitis C virus

**DOI:** 10.1038/emi.2014.19

**Published:** 2014-03-26

**Authors:** Stephanie Pfaender, Richard JP Brown, Thomas Pietschmann, Eike Steinmann

**Affiliations:** Institute for Experimental Virology, Twincore Centre of Experimental and Clinical Infection Research; a joint venture between the Hannover Medical School and the Helmholtz Centre for Infection Research, Hannover 30625, Germany

**Keywords:** genetic diversity, hepacivirus, hepatitis C virus, homologs of hepatitis C virus, pegivirus

## Abstract

Hepatitis C virus is considered a major public health problem, infecting 2%–3% of the human population. Hepatitis C virus infection causes acute and chronic liver disease, including chronic hepatitis, cirrhosis and hepatocellular carcinoma. In fact, hepatitis C virus infection is the most frequent indication for liver transplantation and a vaccine is not available. Hepatitis C virus displays a narrow host species tropism, naturally infecting only humans, although chimpanzees are also susceptible to experimental infection. To date, there is no evidence for an animal reservoir of viruses closely related to hepatitis C virus which may have crossed the species barrier to cause disease in humans and resulted in the current pandemic. In fact, due to this restricted host range, a robust immunocompetent small animal model is still lacking, hampering mechanistic analysis of virus pathogenesis, immune control and prophylactic vaccine development. Recently, several studies discovered new viruses related to hepatitis C virus, belonging to the hepaci- and pegivirus genera, in small wild mammals (rodents and bats) and domesticated animals which live in close contact with humans (dogs and horses). Genetic and biological characterization of these newly discovered hepatitis C virus-like viruses infecting different mammals will contribute to our understanding of the origins of hepatitis C virus in humans and enhance our ability to study pathogenesis and immune responses using tractable animal models. In this review article, we start with an introduction on the genetic diversity of hepatitis C virus and then focus on the newly discovered viruses closely related to hepatitis C virus. Finally, we discuss possible theories about the origin of this important viral human pathogen.

## INTRODUCTION

Hepatitis C virus (HCV) infection is a major cause of chronic liver disease. Currently, about 160 million individuals are persistently infected with HCV.^1^ Acute HCV infection is asymptomatic in many cases, but 50%–80% of infected individuals are unable to clear the virus and a state of persistent viral replication and hepatic inflammation, i.e., chronic hepatitis C, follows. Some patients remain asymptomatic, but over years or decades others develop cirrhosis, portal hypertension, deteriorating liver function and hepatocellular carcinoma. It is due to these late complications that chronic hepatitis C is a leading cause of liver-related death and the prime indication for liver transplantation worldwide.^2^ Combination treatment with pegylated interferon-α and ribavirin has been the standard of care for more than a decade.^3^ Recently, the first directly acting antivirals targeting the viral NS3-4A protease were licensed and triple therapy has further improved treatment options for genotype 1.^4^ However, resistance development, side effects and viral genotype-specific efficacy of these drugs require alternative antiviral treatment options. The HCV genome is 9.6 kb in size, encodes for a single polyprotein that is cleaved by cellular and viral proteases into at least 10 different proteins: the structural proteins core, E1, E2, the ion channel p7 and the non-structural proteins NS2, NS3, NS4A, NS4B, NS5A and NS5B.^5^ These viral factors act in concert with host proteins to mediate virus entry and to coordinate RNA replication and virus production which is coupled to lipoprotein biogenesis.^6^

Ongoing transmission of productive HCV infection is limited to human populations, although higher primates are susceptible to experimental infection.^7^ Due to this narrow host range, a robust immunocompetent small animal model is still lacking. However, a recent study demonstrated infection, replication and *de novo* virus production in an inbred mouse strain engineered to express human HCV entry factors in the liver and carrying a targeted lesion within innate immune signalling genes (*Stat1*, *Irf7*, *Irf9*, *IFN-αβR*).^8^ Although not fully immunocompetent, this landmark study shows that HCV can be propagated in mice and raises the hope that more robust models ultimately with fully functional innate and adaptive immune system can be developed. Despite the rapid advancements in HCV research over the past decade, the origin of HCV still remains elusive, with no evidence of an animal population that might have transmitted the virus to humans.^9^ Nevertheless, the idea that there might be a non-human primate source for HCV infections, as described for HIV,^10^ has engaged researchers since the discovery of HCV in 1989.^11^ The recent discovery of novel hepaciviruses in different animals could now reveal new insights in the origin of HCV. Several reports identified new homologs of HCV in mammals that cluster in the genus hepacivirus or the newly proposed genus pegivirus. Non-primate hepaciviruses (NPHV) were initially discovered in domestic dogs and subsequently in horses^12,13^ and other diverse and widespread HCV-like viruses have been reported in wild populations of rodents and bats.^14,15,16^

## HCV GENETIC DIVERSITY AND CLASSIFICATION

HCV is an enveloped, positive-stranded RNA virus belonging to the genus hepacivirus in the family of *Flaviviridae*. A second species tentatively assigned to this genus is GB virus B (GBV-B), a virus distantly related to HCV identified in a laboratory-housed tamarin, a New World monkey and causing hepatitis.^17^ However, GBV-B infection has not been reported in any other tamarin or other New World monkey species and its origin, as well as its natural host, remains unknown. Besides hepaciviruses, the *Flavivridae* family consists of three other genera: flaviviruses including yellow fever and dengue virus; pestiviruses including bovine viral diarrhea virus and classical swine fever virus and the recently assigned pegivirus genus. Pegiviruses encompass the previously unclassified GB virus A (GBV-A) which was found in primates,^18^ GB virus C (GBV-C) or hepatitis G virus (HGV) which infects humans and chimpanzees^19,20^ and GB virus D (GBV-D) identified in bats.^21^ Of note is that in contrast to the hepaciviruses, GBV-A and some GBV-C/HGV isolates do not appear to encode a core protein.^22,23,24^ On the basis of phylogenetic relationships, observed similarities in genome organization and pathogenic features Stapleton and colleagues proposed this new genus within the family of *Flaviviridae.*^25^

**Figure 1:**
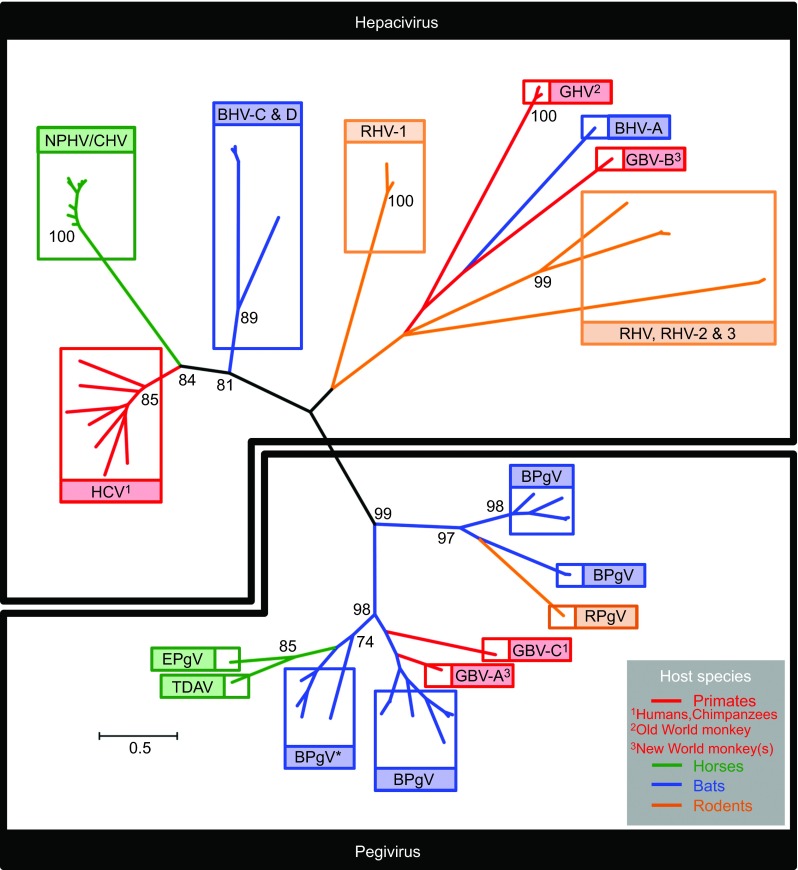
Phylogenetic analysis of the NS3 protease domain of diverse members of the hepaciviruses and pegiviruses (Table 2), with susceptible host species incorporated. Radial tree topology highlighting distinct viral clades (boxed) within the hepacivirus (top) and pegivirus (bottom) genera. Viral clades are color-coded according to susceptible host species (primates: red, horses: green, bats: blue and rodents: orange). Phylogeny was generated using 54 nucelotide sequences encoding a 534-bp fragment at the N-terminus of the *NS3* gene, corresponding to the virally encoded protease domain (genome coordinates 3420–3954 HCV genotype 1a strain; accession number NC_004102). Sequences were aligned according to overlying amino acids using the Clustal W algorithm implemented in MEGA5, and gap-stripped prior to tree construction. Tree was generated using the maximum likelihood method implemented in MEGA5 under the GTR+I+Γ model of nucleotide substitution. Values assigned to deep internal nodes within the phylogeny represent bootstrap supports for groupings. Values presented are percentages, derived from 1000 replications, with only significant values shown (>70%). The pegivirus grouping and the deep divergences within it are generally well supported, indicating the monophyly of the genus. The grouping of HCV, NPHV/CHV and BHV-C and D within the hepacivirus genus is also well supported. However, the deep divergences apparent between the remaining members of the genus are characterised by extremely long connecting branch-lengths and non-significant bootstrap values. This is indicative of ancient divergence between members, in addition to sparseness of taxon sampling: it is likely that many hepaciviruses in this portion of the tree remain either undiscovered, or have become extinct along with their hosts. Branch lengths are in accordance with the scale bar and are proportional to nucleotide substitutions per site. BPgV* clade containing the virus formerly designated GBV-D. Bat hepacivirus, BHV; bat pegivirus, BPgV; rodent hepacivirus, RHV; rodent pegivirus, RPgV; guereza hepacivirus, GHV; Theiler's disease-associated virus, TDAV; equine pegivirus, EPgV.

HCV is genetically highly variable and, based on phylogenetic analyses, viral isolates are grouped into seven genotypes (1–7) (Figure 1), which are associated with specific global regions and modes of transmission.^26^ The high observed genetic diversity is a result of the error-prone replication of the virally encoded RNA dependent RNA polymerase, coupled with the high replication rate *in vivo.*^27^ This genetic variability exhibited by HCV facilitates immune evasion and contributes to viral persistence. HCV genotypes differ from each other by 30%–35% at the nucleotide level. However, this genetic heterogeneity is not evenly distributed throughout the viral genome, and is concentrated in regional hot-spots. The greatest levels of diversity are observed in the *E1E2* genes encoding the envelope glycoproteins E1 and E2, while other genome regions, such as those encoding the core protein or the helicase domain of the NS3 protein, exhibit higher levels of conservation.^28^ Despite substantial sequence variation, all genotypes share the same genome structure, encoding a single polyprotein which is postranslationally cleaved by viral and host proteases into 10 mature proteins. The single open reading frame (ORF) is flanked by two highly conserved untranslated regions (UTRs). HCV genotypes can be further divided into multiple distinct subtypes (a, b, c, *etc.*) differing by 20%–25% at the nucleotide level.^29^ Even within an infected patient, HCV exists as a heterogeneous population of genetically distinct yet related variants.^30^ The prevalence of HCV genotypes differs significantly in different parts of the world. Seventy percent of patients in North America and Europe are infected with genotype 1, while HCV genotypes 2 and 3 account for about 25% of cases, but are overrepresented among patients who inject drugs.^31^ In contrast, HCV genotype 3 can be detected in about 65% of patients in South East Asia and genotype 4 is the major genotype in the Middle East and northern Africa. Genotypes 5 and 7 have been detected mainly in Africa, while genotype 6 has been found in China and Southeast Asia.^29^ Phylogenetic analyses, coupled with global distribution patterns of HCV genotypes, indicate that genotypes 4 and 6 emerged CA 350–700 years ago.^32^ The worldwide dissemination of genotype 2 began later some 90–150 years ago, with the restricted diversity of sequences among genotype 1b indicating emergence around 60–70 years ago.^33^ One has to keep in mind that this molecular clock analysis might be under- or overestimated due to mutational masking and substitutional saturation.

## NOVEL HEPACIVIRUSES IN DIFFERENT ANIMALS

### Dogs/horses

**Figure 2:**
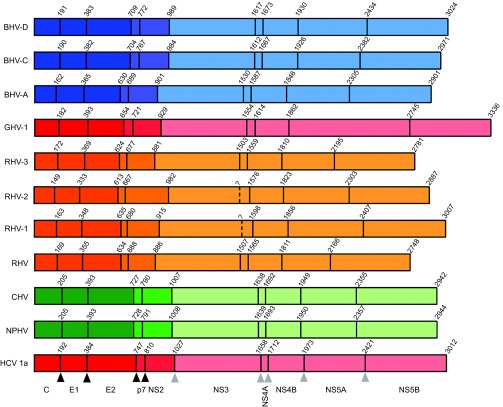
Reference cartoons depicting polyprotein organisation and cleavage site coordinates for distinct hepaciviruses. Cartoons of recently described hepaciviral polyproteins relative to an HCV reference (bottom) are shown, depicting locations of homologous protease cleavage sites, with names of represented hepaciviruses located to the left of each polyprotein schematic. Coordinates located above each cartoon represent the first residue of each encoded protein. The 10 cleaved proteins are detailed below the schematic cartoon of the HCV genotype 1a reference polyprotein. Black and grey triangles located below the HCV cartoon indicate host and viral protease cleavage sites, respectively. Peptide cleavage sites have been experimentally validated for HCV, while recently identified hepaciviral homologs have predicted cleavage sites which require further experimental validation. Color coding according to susceptible host species is identical to Figure 1. Structural proteins (core, E1 and E2) associated with infectious virions are shaded dark, non-structural proteins essential for assembly (p7 and NS2) are shaded intermediate and non-structural proteins associated with the replication module (NS3, NS4A, NS4B, NS5A and NS5B) are shaded light. GenBank accession numbers for representative strains are HCV-1a: NC_004102, NPHV: JQ434008, CHV: JF744991, RHV: KC815310, RHV-1: KC411777, RHV-2: KC411784, RHV-3: KC411807, BHV-A: KC796077, BHV-C: KC796090 and BHV-D: KC7960074.

Utilizing both deep sequencing and serology-based approaches, a number of novel hepaciviruses have been identified in different mammalian host species. Kapoor and colleagues^13^ were the first to discover a canine homolog of HCV which was phylogenetically closely related to HCV. Initially termed canine hepacivirus (CHV), the virus was independently sequenced from dogs with a respiratory illness from two different outbreaks of respiratory disease in the United States, whereas no healthy animals were found to be infected. High viral loads (>10^7^ viral copies/nasal swab) were detected in nasal swabs of the infected animals. Testing liver and lung samples from 19 unrelated dogs which had died of unexplained gastrointestinal illness, CHV RNA was found at low levels (<10^3^ copies/2 ng of total RNA) in liver samples, although lung tissue contained no detectable viral RNA. Furthermore, using *in situ* hybridization, Kapoor *et al.* revealed focal and dispersed infection of canine liver and the presence of viral RNA mainly in the cytoplasm of hepatocytes. Strikingly, there was a high degree of genetic relatedness between viral sequences from different animals which is rather unexpected for a RNA virus. The viral genome of CHV encodes a 2942 amino acid (aa) polyprotein, which is predicted to be cleaved into 10 mature viral proteins (Figure 2), in a similar fashion to that described for HCV^5^ (Table 1). Comparative phylogenetic analyses of conserved regions of the predicted helicase (NS3) and RNA-dependent polymerase (NS5B), revealed CHV to be the closest relative of HCV discovered to date, and equidistant from all seven HCV genotypes (Figure 1). The virus displayed approximately 50% nucleotide sequence divergence from HCV with a maximum aa identity to HCV in the non-structural proteins NS3 and NS5B (>55%–65%), whereas E1, the N-terminal half of E2, NS2 and the C terminus of NS5A showed the lowest aa identity (<35%–45%). Surprisingly, the glycoprotein regions of CHV were readily aligned with HCV, with marked similarity in the C-terminal half of E2. Analyses of the E1/E2 glycosylation sites revealed 4 and 10 potential potential *N*-linked glycosylation (PNG) sites, respectively, comparable to those predicted for HCV (E1 4 or 5 and E2 up to 11).^34^ PNG sites are essential for immune shielding, efficient entry and correct folding of the HCV envelope glycoproteins incorporated into infectious virions.^35^ Intriguingly, the binding site for miR-122, which is important for HCV replication, in the 5′ UTR could not be identified in the CHV sequence. Together with the absence of microRNA sequences in the dog genome capable of binding to the equivalent site in CHV, this finding argues that this virus might not depend on miR-122 for its replication. The mean time to the most recent common ancestor between the HCV genotypes and CHV was estimated to be between 500 and 1000 years before present (Ybp).

In an effort to further investigate the host range of CHV, the same group utilized a serology-based approach to screen for the presence of the virus in other mammalian species.^12^ Burbelo and colleagues used a recombinant protein expressed from the helicase domain of CHV NS3 as antigen to detect CHV antibodies in sera from different animals. Surprisingly, they detected immunoreactivity against CHV NS3 in 36 samples of 103 horses (3%), with eight horse sera also carrying viral RNA, whereas 80 dogs, 14 rabbits, 81 deer and 84 cows were seronegative, with one intermediate positive sample from a cow. Initial sequencing of the eight viral RNA positive horse samples identified a series of genetically diverse viruses.^12^ Based on these findings in a different host, the authors tentatively termed these viruses NPHV.^12^ Complete genomic sequences from these eight NPHV variants were obtained (Table 2). The original CHV variants showed a very high similarity (maximum of 0.35% divergence) to one of the eight NPHV variants, whereas the eight horse-derived NPHV sequences themselves had moderate genome sequence diversity from each other (6.4%–17.2%). This high degree of homology between NPHV and CHV combined with the lack of other hepaciviruses in dogs suggests that the CHV isolate may in fact be a horse virus that was transmitted to a dog. If that was a true transmission event or simply a contamination, possibly by feeding on horse meat or veterinary products which utilized horse based components is unclear. At the aa level, the structural region showed a greater divergence than the non-structural region. Of note, most sequence diversity between NPHV variants was attributable to synonymous substitution (*D*_S_), with low levels of non-synonymous substitution (*D*_N_) variation observed. Indeed, low *D*_N_/*D*_S_ ratios of between 0.03 and 0.05 indicate the action of strong purifying selection on NPHV genomes when compared to HCV, which displays a higher frequency of *D*_N_ variation and thus higher *D*_N_/*D*_S_ ratios. In general, sequence divergence among NPHV isolates was greater than subtype diversity within HCV. Even though the originally predicted CHV secondary structure of the 5′ UTR showed occlusion of both miR-122 binding sites, in the revised NPHV 5′ UTR, the second miR-122 seed site was both open and completely conserved, implying a possible hepatotropism of NPHV as observed for HCV (Table 1). Unlike HCV, which chronically infects 50%–80% of exposed humans, only 22% of horses showed co-presence of IgG antibodies and viral genomes. This could be indicative for either an acute infection or would suggest that possibly the majority of equine hosts are able to clear NPHV infection.

To further investigate the species specificity of NPHV and to address questions regarding tissue tropism or pathogenesis of this newly identified hepacivirus, the group of Simmonds and colleagues analyzed NPHV in domestic horses in the United Kingdom.^36^ Screening for the presence of NPHV in other mammals, the authors performed large-scale polymerase chain reaction (PCR)-based investigation of samples from dogs, cats, pigs, rodents, donkeys and horses. From this survey, three plasma samples from 142 horses (2%) were found positive for NPHV RNA. Sequence comparison demonstrated that each of the positive horses was infected with NPHV variants distinct from the eight previously identified in horses. The newly identified NPHV variants displayed similar branching orders in each genome region, indicating a lack of recombination as observed previously.^12^ Clinical records from the time of sampling displayed no evidence for a hepatic or systemic disease. Furthermore, liver function analyses revealed no indication for hepatic inflammation as γ-glutamyl transferase and glutamate dehydrogenase values were within reference range, with the exception of a mildly elevated γ-glutamyl transferase level in one horse. Moreover, with the bile acid levels within reference range, there was no sign of hepatic damage. Repeated samplings from one horse four and five months after the initial sampling revealed a persistent infection. The horse remained viremic, but the viral load decreased between the fourth and fifth months from the initial 4.8×10^7^ copies/mL to 2.1×10^5^ and 7.1×10^4^ copies/mL, respectively. During the sampling period, the horse remained clinically unremarkable and the liver indices stayed mainly within the reference ranges, although frequently at the upper end of the reference ranges. This data indicate that hepaciviral infections in horses can be persistent. The organ tropism of NPHV has yet to be elucidated and further studies on viral associated pathogenesis, the course of clinical disease and likely mode(s) of transmission are required to fully understand the nature of NPHV infection in horses.

### Rodents/bats

The discovery of closely related hepaciviral homologs of HCV, which naturally infect equine hosts, raised the possibility that additional novel hepaciviruses may infect a range of mammalian hosts. Therefore, Kapoor and colleagues^14^ initiated a search for such viruses in several species of wild rodents. PCR screening of plasma samples from over 400 wild-caught rodents, using degenerate primers targeting conserved helicase domains of both hepaciviruses and pegiviruses, uncovered evidence for hepaciviruses and pegiviruses in 18 samples of rodents belonging to four species: hispid pocket mice (*Chaetodipus hispidus*), deer mice (*Peromyscus maniculatus*), desert wood rats (*Neotoma lepida*) and white-throated wood rats (*Neotoma albigula*). The authors tentatively named these viruses rodent hepacivirus (RHV) and rodent pegivirus (RPgV) according to the guidelines of the International Committee for Taxonomy of Viruses. The complete genome of one RHV variant (RHV-339) and the nearly complete genome of another variant (RHV-098) were acquired from plasma samples originating from two different deer mice (Table 2). Based on these sequences, the RHV genome is predicted to encode a polyprotein of 2748 aa flanked by 5′ and 3′ UTRs (Table 1). In one of the sequenced variants (RHV-339), a putative miR-122 seed site was apparent in the 5′ UTR, suggesting a possible dependence on miR-122 of these novel viruses. The polyprotein is predicted to encode 10 proteins which were similar in predicted size to those of HCV and other hepaciviruses (Figure 2). The RHV glycoproteins E1 and E2 contained two and four PNG sites, respectively, considerably less than those observed for HCV and NPHV. The computed genetic distance between RHV-339 and HCV in the structural regions ranged from 67% to 77% and 65% to 70% in the non-structural regions, and was therefore substantially greater than that between HCV and NPHV.

Additionally, another group independently described novel hepaciviruses infecting rodent hosts.^16^ Drexler and colleagues screened for bloodborne viruses in sera and organs from 4770 rodents and sera from 2939 bats. Even though bat serum showed cross-reactivity with HCV antigens, no hepacivirus RNA was detected in these samples. Nevertheless, the group identified three highly divergent novel RHV clades in European bank voles (*Myodes glareolus*) and in South African four-stripped mice (*Rhabdomys pumilio*). To determine the genome organization and structural features of RHV, near full genomes of five representative hepaciviruses from all rodent clades were determined (Table 2). The five sequenced RHV genomes were predicted to encode a typical hepacivirus polyprotein comprising three structural and seven non-structural proteins of comparable size to those previously described for other hepaciviruses (Figure 2: RHV-1, 2 and 3). Again, PNG sites in the envelope proteins, in particular in the putative E2 protein, were predicted to be fewer when compared to HCV (Table 1). The minimum aa identity of the novel rodent viruses to HCV averaged in the structural proteins between 15.1% and 31.3% and in the non-structural proteins from 16.3% to 42.2%. Based on the high degree of nucleotide sequence homology of the *NS5B* genes between all members of the *Flaviviridae* family, comparative analysis was possible and revealed the grouping of RHV as a monophyletic sister-clade to HCV. Interestingly, all RHV clades were slightly more related to GBV-B than to HCV (Figure 1). Further genomic analyses revealed the presence of one miR-122 binding site, again pointing to a possible hepatotropism for these viruses. Indeed, the authors were able to find high viral loads in bank vole tissues infected with RHV, with the highest RNA concentrations found in liver tissue: 1.8×10^8^ copies/g compared to significantly lower concentrations in lung, kidney, serum, spleen, heart, brain and intestine. *In situ* hybridization revealed foci of viral RNA in the cytoplasm of *M. glareolus* hepatocytes, whereas no evidence of virus infection by *in situ* hybridization could be found in the other organs. Evidence for liver inflammation was observed by histopathological examinations, which revealed low-grade focal lymphatic invasion in the RNA-positive animals compared to the RNA-negative animals. Serological investigations of the *Myodes* hepaciviruses revealed the presence of antibodies against the NS3 antigens of these viruses in only some of the animals (8.3% and 12.4%), and no cross-reactivity of the sera with HCV could be observed. This indicates a specific immune reaction against RHV. RNA and antibodies were found in 3 of 57 (5.3%) PCR-positive bank vole sera and this low co-occurrence could indicate that bank voles might be able to clear hepacivirus infections in the majority of cases. The identification of rodent homologs of HCV could pave the way for novel surrogate animal models of HCV which may ultimately facilitate vaccine design and treatment approaches.

Although the group of Drexler and colleagues failed to directly recover hepacivirus genomes in bats, they were able to show serological evidence for these viruses in bats, suggesting the existence of bat hepaciviruses (BHVs). Indeed, the group of Quan and colleagues^15^ identified bats as a major natural reservoir for hepaciviruses and pegiviruses. The group utilized an unbiased high-throughput sequencing approach to enhance the knowledge of viral diversity in bats and encountered a highly diverse group of bat-derived viruses which were related to hepaciviruses and pegiviruses. The group was able to detect viral genomes in six of the eight bat families tested and in a total of 78 sera/plasma samples, one lung specimen as well as one rectal swab. The viral load was determined by quantitative PCR and ranged from 10^3^ to 10^8^ RNA copies/mL in the sera or plasma of infected bats. Taken together, the group was able to identify 83 bat-derived viruses, which potentially represent 22 novel viral species. The BHVs fell into three highly divergent clades exclusively composed of viruses from two species of African bats (*Hipposideros vittatus*, *Otomops martiensseni*). Clade A viruses were most closely related to GBV-B whereas clade C and D viruses fell into a basal position relative to the clades containing NPHV and HCV. BHV serum levels ranged from 1.07×10^5^ to 3×10^8^ RNA copies/mL. Five near full-length genome sequences were obtained representing all three clades of BHV (Table 2). BHV genomes encode a single positive-stranded RNA genome containing a single ORF, flanked at the 5′ and 3′ end by non-translated regions, encoding a polyprotein precursor of about 2901 to 3024 aa (Figure 2). Consistent with other viruses in the family of *Flaviviridae*, conserved protein domains were recognized in the predicted polyprotein. Comparable to other hepaciviruses, the most variable regions of the genome encode the envelope glycoproteins, the non-structural proteins NS2 and NS5A with less than 34.5%, 33.5% and 20.4% aa sequence identity, respectively. The most conserved regions were the NS3 and NS5B proteins, with aa sequence identities of 39.2%–53.3% in the *NS3* gene and 35.3%–46% in the *NS5B* gene. Again, the translated BHV E1 and E2 protein sequence showed fewer PNG (6–7) sites compared to HCV (Table 1). Regarding pathogenesis of these new viruses, further investigations are required. Even though there were high levels of viremia detected, all bats collected were apparently healthy, suggesting that BHVs may not be pathogenic to their host. Bats are probably the most abundant, diverse and geographically dispersed vertebrates worldwide.^38^ The discovery of BHV and bat pegivirus (BPgV) in bats adds new members to the ever expanding list of viruses that can infect this animal reservoir. Many different zoonotic viruses, including rabies virus and related lyssaviruses, Nipah and Hendra viruses as well as Ebola and severe acute respiratory syndrome coronavirus and recently also hepadnaviruses have been found to originate in bats.^39,40^ Further research might shed new light on the role of these mammals as carriers of members of the *Flaviviridae* and may provide new insights into the evolutionary origins of HCV.

### Non-human primates

Initially, novel hepaciviruses were all identified in non-primate species. Recently, the group of Lauck and colleagues expanded this host range further with the discovery and characterization of the first hepacivirus infecting a wild non-human primate, the black-and-white colobus (*Colobus guereza*), an Old World monkey from Uganda.^37^ Notably, all infected animals appeared healthy at the time of sampling with no observed overt clinical symptoms. Deep sequencing of RNA from plasma samples of nine of these animals revealed the presence of viral RNA in three animals, with the genomic architecture matching other viruses from the hepacivirus genus. The virus, named gnereza hepacivirus (GHV), was shown to share a common ancestry with GBV-B, the recently identified RHVs and one of the three recently discovered BHV clades (clade A). Viral sequences covering the entire coding region as well as partial 5′ and 3′ UTRs were obtained from all three animals. The new viruses share limited nucleotide identity across the coding region with other known hepaciviruses (HCV 43%, NPHV 43%, RHV 47%, GBV-B 48% and BHV 50%). Based on sequence comparison, two highly similar variants were classified as subtype GHV-1, whereas the third variant represents subtype GHV-2. Interestingly, a canonical miR-122 binding site was present in both GHV subtypes. The predicted cleavage sites of the GHV polyprotein are similar to other hepaciviruses, with ten mature viral proteins predicted. The glycoproteins E1 and E2 contain four PNG sites each (Table 1). A striking feature of theses hepaciviruses is their unusually long *NS5A* gene (882–883 aa), approximately twice the length of any other known *NS5A* within the *Flaviviridae*, rendering the genomic sequence over 1 kb longer than that of HCV (Figure 2). Sliding-window analyses of aa similarities between GHV and other members of the hepaciviruses revealed that across the region from core to NS4B GHV is most similar to BHV, whereas GHV NS5B shares the highest sequence similarity with RHV. This suggests that cross-species transmission and ancient recombination might have contributed to the evolution of GHV. Phylogenetic analyses support the grouping of GHV within the hepacivirus genus (Figure 1) and the shared common ancestry of GHV, GBV-B, RHV and BHV-112. Analyses to determine the most recent common ancestor for GHV, HCV and NPHV suggests an early divergence of GHV from the other hepacivirus lineages, at least 1000–1500 Ybp.^37,41^ Taken together, the detection of GHV represents the first documented natural infection of a non-human primate with a hepacivirus and further expands the known host range of this viral genus.

## NOVEL PEGIVIRUSES IN NEW HOSTS

As a newly proposed genus of the family of the *Flaviviridae*, the genus pegivirus has been proposed to encompass the so far unclassified viruses GBV-A, GBV-C/HGV and GBV-D.^25^ Based on this new classification, different groups also identified multiple viruses falling into the pegivirus genus, which are emerging in new hosts. Chandriani and colleagues^42^ identified a highly divergent member of the *Flaviviridae* family, which is likely the causative agent for an outbreak of acute hepatic disease occurring on a horse farm. The virus was present in equine serum from two overtly clinical index cases of hepatitis and from an equine plasma product which was administered to the horses before the outbreak. This new pathogen was designated Theiler's disease-associated virus (TDAV), presumably the causative agent of Theiler's disease, an acute hepatitis in horses. Viral titers in most of the infected horses ranged from 10^7^ to 10^8^ genomes/mL. The viral genome contained a single ORF encoding a polyprotein of 3189 aa flanked by a 5′ and 3′ UTR. Based on comparison with related members of the *Flaviviridae*, the virus is predicted to encode three structural proteins (core, E1 and E2) and seven putative non-structural proteins (a protein between E2 and NS2 as well as NS2, NS3, NS4A, NS4B, NS5A and NS5B). Phylogenetic analyses grouped TDAV as belonging to the newly proposed pegivirus genus (Figure 1). Over the length of the entire polyprotein, the virus shares 35.5% aa identity with GBV-D, 20.5% identity with HCV (genotype 1) and 20.4% identity with NPHV. The most conserved regions can be found in NS3 and NS5B, with regions exceeding 75% aa identity with GBV-D. Interestingly, even though TDAV is linked to an acute hepatitis, no miR-122 binding site could be identified, suggesting that, unlike HCV, the virus can replicate independently of miR-122. Transmission between horses appears to be low as the virus was undetectable in horses which had contact with other infected animals. The TDAV-positive horses were further ranked as clinical or subclinical, with the clinical cases displaying significantly elevated liver enzymes in serum. The subclinical cases displayed varying degrees of elevated liver enzymes in the serum, but without overt clinical manifestation of liver disease in most cases. In general, a slightly higher proportion of asymptomatic TDAV-positive animals were observed. Furthermore, a clear relationship between viral load and clinical hepatitis could not be observed, indicating that the viral load was not predictive of the extent of hepatic injury. Further analyses of the infected horses 1 year after the outbreak provided evidence that TDAV can establish a chronic infection. To further investigate the route of transmission for TDAV infections, the authors performed an inoculation study in which they experimentally injected four healthy animals with a TDAV-positive plasma product. Even though the viral genome could be detected in all inoculated horses, only one horse showed a clear elevation of liver enzymes over the course of the study. In summary, this study provides evidence for the association of TDAV with Theiler's disease, identifying TDAV as the sole member of the newly described genus pegivirus for which disease association has been demonstrated.

Kapoor and colleagues^43^ independently identified another equine pegivirus (EPgV) which is genetically distinct to TDAV. The group screened serum samples from horses with elevated liver enzyme levels and detected viral RNA in two samples, which were highly divergent from all previously known pegiviruses (Table 3). The virus encodes a 5′ UTR, a single ORF encoding a putative polyprotein of 3305 aa and a 3′ UTR. Sequence alignments revealed the divergence of EPgV from other pegiviruses, ranging from 62% to 77% in the structural genes and from 49% to 59% in the non-structural region. The most conserved regions were the *NS3* and *NS5B* genes, as described for the hepaciviruses genus, whereas the highest sequence diversity can be found in the glycoproteins E1/E2 and NS4B. To analyze the prevalence and persistence of EPgV, two horse cohorts were studied. In one cohort, the viremia frequencies were 25% (3/12) among horses with elevated liver enzymes and 6.4% (4/62) among healthy animals. In another herd analyzed at different time points over four years, 15%–32% of the horses were found to be positive, with two horses remaining viremic for at least three and a half years and two horses being able to clear the infection. Viral genome copy numbers ranged from 10^4.5^ to 10^6.5^ genome equivalents/mL in the serum and viral RNA could also be detected in liver and lymph node biopsy samples, as well as in peripheral blood mononuclear cells, with no major differences in viral RNA between the tissues, suggesting that the virus is not strictly hepatotropic.^43^ Further studies are necessary to determine the prevalence, tissue tropism and possible disease association of this newly identified virus.

The same group additionally identified two new species of pegiviruses in rodents (see the section on ‘Rodents/bats'), one from white-throated wood rats (RPgV-cc61) from which the whole genome was acquired (Table 3) and the other from deer mice.^14^ The viral genome displayed the typical *Flaviviridae* genome organisation including a 5′ UTR, a polyprotein coding region (3484 aa) as well as a 3′ UTR. Genetic analyses revealed that RPgV was substantially divergent from GBV-A, GBV-C, GBV-D and from the EPgV (Figure 1). Amino-acid divergence ranged from 78% to 81% in the structural proteins and 54% to 56% in the non-structural region.

Apart from rodents, bats comprise the most diverse group of mammals. The *Pteropus giganteus* bats have already been identified as carriers of GBV-D.^21^ The group of Quan and colleagues further enhanced our understanding of the viral diversity in bats, with the identification of bat-derived hepaciviruses and pegiviruses^15^ (see the section on ‘Rodents/bats'). Among the total of 83 bat-derived viruses uncovered, 19 potential novel viral species within the pegivirus genus were identified, which formed three distinct lineages. Clade H viruses clustered with the previously identified GBV-D, whereas the clade G and K viruses formed distinct phylogenetic clusters within the genus pegivirus. Interestingly, in some of the animals, coinfections of clade G and K viruses and in one case even coinfection with a hepacivirus (clade C) and a pegivirus (clade K) were observed. The genomic organization of these pegiviruses is similar to that observed for other flaviviruses. Amino acid sequence identities were greatest in non-structural proteins NS3 and NS5B, whereas the envelope proteins as well as NS2 and NS5A displayed the highest variability. In summary, these studies show that also pegiviruses are widely distributed among different mammalian species and future studies are necessary to investigate disease association, transmission and tissue tropism.

## THEORIES FOR THE ORIGIN OF HCV

Viruses are usually well adapted to their hosts, resulting in multiple barriers to cross-species transmission.^44^ Nonetheless, the majority of recent emerging infections in human populations represent zoonoses from wild animal species.^45^ A recent survey, using fruit bats as a model organism, uncovered 55 novel viruses from seven viral families.^46^ Extrapolating this number to all mammalian species, the authors estimate a minimum of 3.2×10^5^ mammalian viruses awaiting discovery. Although this number is likely to be an overestimation due to the tendency of bats to act as reservoirs for a broad spectrum of viruses, it is clear that many as yet undiscovered viruses circulate in wild animals, all with the potential to jump the species barrier to humans. Cumulatively, these findings would support a scenario where HCV in humans is likely the result of a cross-species transmission, probably from an as yet unidentified source.

Current patterns of global HCV diversity could be due to a single ancestral zoonotic transmission to archaic humans prior to their global dispersal, with subsequent viral diversification in isolated populations. Identification of a more closely related hepaciviral progenitor closer to the root of extant circulating HCV isolates than NPHV, the current closest relative, would provide support for this scenario.^47^ However, the geographical associations of different HCV genotypes, which appear to have been evolving and diverging in geographically discrete human populations over an extended time scale, could also be the result of multiple, more recent, independent cross-species transmissions to geographically separated human populations. Identification of novel hepaciviruses, isolated from animal hosts, which fall within the global HCV radiation and are positioned basally to distinct HCV clades, would ultimately provide strong evidence for this alternative.^47^

The >1200 bat species are known to harbour a diverse array of viruses from multiple families, many of which have crossed the species barrier to cause a variety of diseases in humans, often via an intermediate host.^48^ For example, the Hendra virus, an RNA virus from the family *Paramyxoviridae*, has caused multiple outbreaks in horses and four reported human fatalities.^49^ Interestingly, Hendra virus was subsequently demonstrated to originate in fruit bats and was transmitted to humans via close contact with horses. Of note, the most closely related animal hepaciviruses to HCV described to date are found in horses (NPHV)^12^, and Kenyan fruit bats (BHV)^15^ (Figure 1). Thus, if continued wild animal sampling fails to identify more likely candidates for the ancestor(s) of HCV, one could envisage an historical scenario whereby the progenitor of HCV was transmitted to humans from bats via horses, as all three species are susceptible to hepaciviral infection. However, viruses are dependent on a multitude of host factors to complete their life cycle in susceptible cells. Thus, species-specific barriers to viral cross-species transmissions are likely to be proportional to the genetic relatedness of host species: one would expect viruses to jump the species barrier more easily between closely related host species. In addition to HCV infection of humans, GBV-B infection of New World monkeys and the recent discovery of distantly related hepaciviral homologs to HCV in Old World monkeys^37^ indicates the susceptibility of a broad array of primates to hepaciviral infection. Two of the biggest infectious disease burdens afflicting humanity presently are HIV and Malaria, zoonoses from chimpanzees and gorillas, respectively,^50^ our closest great-ape relatives. Thus, it might be also possible that great apes, or other primate species, harbor as yet undescribed hepaciviruses, which may ultimately have given rise to the current HCV pandemic.

## CONCLUDING REMARKS

Despite successful development of cell culture systems to study the complete HCV replication cycle,^51^ analysis of mechanisms of virus pathogenesis and immune control as well as vaccine development are severely hampered by the lack of robust immunocompetent small animal models. Furthermore, the origin of HCV has remained elusive. Recently, studies combining powerful next-generation sequencing technologies with coordinated sample collection from multiple geographical localities have uncovered related hepaciviral and pegiviral homologs in diverse animal species. These research efforts have resulted in a rapid expansion of the known viral diversity in hepacivirus and pegivirus genera, and identified an increasing number of animal host species susceptible to hepacivirus and pegivirus infection. Differences and similarities between these newly discovered viruses and HCV may ultimately advance our understanding of hepacivirus biology with respect to mechanisms of hepaciviral replication, permissiveness of small animal models to productive infection, epidemiology and vaccine development, in addition to further elucidating HCV origins. Future studies should address transmission and ecology of these new viruses in their natural hosts to evaluate any potential risk of trans-species transmission to humans. The generation of functional cDNA clones will further advance our knowledge on hepaciviral replication strategies and could be used for the development of recombinant HCV vaccines. In conclusion, the recent discoveries of multiple novel hepaciviruses in diverse mammalian species have undoubtedly intensified research efforts to identify the true origins of HCV. Ultimately, these studies will expand our knowledge of circulating mammalian viruses, in addition to further illuminating barriers/pathways to viral cross-species adaptation.

## Figures and Tables

**Table 1:** Comparison of features from novel hepaciviruses

	HCV^a^	CHV/NPHV	RHV	BHV	GHV
Polyprotein	3011 aa	2942 aa	2748–3007 aa	2901–3024 aa	3334–3336 aa
Number of predicted proteins	10	10	10	10	10
Host of identification	Humans	Dogs/horses	Rodents	Bats	Old World monkeys
Viral RNA detected	Liver	Dogs: respiratory tract/liver; horses: serum	Plasma, serum, organs	Serum	Plasma
Pathogenesis	Viral hepatitis	Horses: apparently healthy, one horse slightly elevated liver enzymes	Evidence for liver inflammation	None detected	None detected
E1 and E2 linked glycosylation sites	4 and 11	4 and 10	2 and 4	2 and 4–5	4 and 4
Bindig sites miR-122	Two miR-122 seed sites	Second miR-122 seed site was both open and completely conserved	One miR-122 seed site	ND^b^	Canonical miR-122 binding site

a Reference strain H77.

b ND, not described.

**Table 2:** The newly discovered hepaciviruses, host species, sequence accession number and isolate designation

Host	Name	GenBank accession number	Isolate	Reference
Dog	CHV	JF744991	AAK-2011	13
Horse	NPHV	JQ434001	NPHV-NZP-1	12
		JQ434002	NPHV-G1-073	
		JQ434003	NPHV-A6-006	
		JQ434004	NPHV-B10-022	
		JQ434005	NPHV-F8-068	
		JQ434006	NPHV-G5-077	
		JQ434007	NPHV-H10-094	
		JQ434008	NPHV-H3-011	
		JX948116	EF369/11	36
		JX948119	EF317/98	
		JX948121	EF330/97	
		KC411810	GER/Eq179	16
		KC411811	GER/Eq285	
		KC411814	GER/Eq105	
		KC411813	GER/Eq120	
		KC411812	GER/Eq143	
Rodent^a^	RHV	NC_021153	RHV-339	14
		KC815312	RHV-098	
	RHV-1	KC411777	RMU10/3382	16
		KC411796	NLR08/365	
	RHV-2	KC411784	NLR/oct70	
	RHV-3	KC411806	SAR-3	
		KC411807	SAR-46	
Bat^a^	BHV clade A	KC796077	PDB-112	15
	BHV clade C	KC796090	PDB-452	
		KC796091	PDB-445	
	BHV clade D	KC796078	PDB-491.1	
		KC796074	PDB-829	
Old World monkey	GHV	KC551800	GHV-1 BWC08	37
		KC551801	GHV-1 BWC05	
		KC551802	GHV-2 BWC04	

a Only viruses listed whose near full-length genomes were generated.

**Table 3:** The newly discovered pegiviruses, host species, sequence accession number and isolate designation

Host	Name	GenBank accession number	Isolate	Reference
Horse	TDAV	KC145265	HorseA1_serum	42
	EPgV	KC410872	C0035	43
Rodent^a^	RPgV	NC_021154	RPgV-cc61	14
Bat^a^	BPgV clade G	KC796080	PDB-1698	15
		KC796087	PDB-1734	
		KC796093	PDB-34.1	
		KC796076	PDB-620	
		KC796084	PDB-76.1	
		KC796079	PDB-99	
	BPgV clade H	KC796088	PDB-1715	
		KC796083	PDB-694	
		KC796073	PDB-303	
	BPgV clade K	KC796082	PDB-24	
		KC796089	PDB-491.2	
		KC796081	PDB-737B	
		KC796075	PDB-106	
		KC796086	PDB-838	
		KC796085	PDB-534	

a Only viruses listed whose near full-length genomes were generated.
